# Anxiety and Depression among Amphetamine-Type Stimulant Users: The Association with Religiosity and Religious Coping

**DOI:** 10.21315/mjms2020.27.4.5

**Published:** 2020-08-19

**Authors:** Chok How Tan, Rusdi Abd Rashid, Ng Chong Guan

**Affiliations:** Department of Psychological Medicine, Faculty of Medicine, Universiti Malaya, Kuala Lumpur, Malaysia

**Keywords:** anxiety, depression, religious, religious coping, amphetamine users

## Abstract

**Background:**

Amphetamine-type stimulant (ATS) use brings severe adverse effects to the mental well-being of an individual and it is an essential contributor to the global disease burden. Meanwhile, religiosity and religious coping might improve one’s conduct, physical and mental well-being. Hence, this study aims to determine the prevalence of anxiety and depression in ATS user and their association with religiosity and religious coping.

**Methods:**

It is a cross-sectional study conducted at the Department of Psychological Medicine, Universiti Malaya Medical Centre, Malaysia. The Hospital Anxiety and Depression Scale (HADS) was used to assess anxiety and depression among ATS users. Religiosity and religious coping were measured with Duke University Religious Index and Brief RCOPE.

**Results:**

This study involved 215 ATS users. Almost half of the ATS users had either anxiety (*n* = 96; 44.6%) or depression (*n* = 108; 50.2%), which were associated with polysubstance use or having an existing psychiatric disorder. Subjects with higher religiosity and positive religious coping were less anxious or depressed. However, negative religious coping was significantly associated with anxiety and depression in ATS users.

**Conclusion:**

Anxiety and depression are prevalent in ATS users. Integrating religiosity and religious coping into the ATS users’ treatment plan helps to improve their mental well-being.

## Introduction

Substance use is a serious public health problem encountered by most of the countries worldwide (1). Amphetamine-type stimulant (ATS) is the second most commonly used substance worldwide (2) and it has been estimated that 55 million people had used ATS during 2016. The number of ATS users in Malaysia has tremendously increased in the past few years. This is evident in the increased number of ATS users that were detained and the amount of ATS seized by the enforcement agencies (3). Although there has been a rapid advancement in other fields of medicine, effective treatments for ATS use are still lacking (4).

ATS users were four times more likely to suffer from any form of psychiatric disorder when compared to a non-ATS user (5). Depression and anxiety commonly occur in ATS users (6, 7), and ATS is associated with lower quality of life and self-harm behaviour (8, 9). When these psychiatric comorbidities are not identified and treated, it can lead to relapse and increased morbidity and mortality in ATS user.

Growing scientific evidence have revealed that religious practices are associated with higher survival rate, better physical health and psychological well-being (12). Multiple studies have also reported that religiosity acts as a good prognosis factor for substance use in both urban and rural areas (13, 14). Religiosity is a sociological term referring to the engagement of a person in religious activities, practices, their intrinsic religious beliefs, faiths, spiritual knowledge and religious identification with his or her religion (15, 16). The construct of religiosity has been of interest to researchers since a century ago when religion seemed to influence one’s conduct, physical and mental well-being in general (17). Numerous studies have also suggested that religious involvement may deter substance use (18, 19). These religious values support a person’s life decisions and the decision to use illicit substance, in particular. Religious involvement has been found to be associated with favourable substance use outcome including a lower usage rate of ATS (20, 21).

Religious coping is referred to as ‘the use of cognitive and behavioural techniques while facing stressful life events which originated from one’s religion or spirituality’ (22). Harrison et al. (23), in their systemic review, reported that religious coping is becoming increasingly researched. They also concluded that religious coping influences one’s physical health, mental health, personality, behaviours and feelings of efficacy. Positive religious coping always linked with better outcome and prognosis in psychiatric patients (24, 25). However, negative religious coping may become a potential stressor on top of their struggles (26). Therefore, religious coping is an essential component of the treatment to overcome anxiety and depression among substance users (23).

Malaysia is a multiracial country consisting of three major ethnic groups: Malay, Chinese and Indian. The national religion is Islam but other religions are freely practised and accepted in this country. The construct, belief and practice of Malaysia population may not be the same based on the existing data from other countries. In addition, to date, no studies have been conducted in Malaysia, examining the association between the role of religion with anxiety and depression among ATS users. As such, the current study aims to assess the religiosity and religious coping among ATS users and their association with anxiety and depression.

## Methods

This was a cross-sectional study conducted in the out-patient psychiatric clinic and in-patient psychiatric ward of the Department of Psychological Medicine, University Malaya Medical Centre, Kuala Lumpur, Malaysia from 1st May 2019 until 30th November 2019. This study was approved by the Medical Research Ethics Committee, Universiti Malaya (MREC ID No: 2019328-7268).

### Participants

Convenient sampling method was used in the study. Patients who visited the out-patient clinic and those admitted to the psychiatric ward were approached and the study was explained. Patients who agreed to participate in the study signed the informed consent forms. Consented patients were then screened for inclusion and exclusion criteria. The inclusion criteria were i) subjects who were diagnosed with amphetamine use disorder using the Diagnostic and Statistical Manual of Mental Disorders, 5th edition (DSM-5); ii) subjects who can understand and read Malay or English; iii) subjects who are above the age of 18 years and iv) subjects who gave consent. The exclusion criteria were i) subjects who acutely psychotic or disturbed and ii) subject who diagnosed with dementia or intellectual disabilities.

### Sample Size

For a cross-sectional prevalence study, the formula (27) that the author used to calculate the sample size was:

$$\begin{array}{l} N={\text{Z}}^{2}\;\text{p}(1\text{p})/{\text{d}}^{2}\\ N=1.96^{2}\;0.168(10.168)/0.05^{2}\\ N\sim 214.78\\ N=215 \end{array}$$

*N* = required sample size
Z = confidence level at 95% (standard value of 1.96)
p = estimated prevalence of depression (the prevalence of depression in ATS dependence user was 16.8%) (28)
D = margin of error at 5% (standard value of 0.05)

Based on the calculation above, total numbers of subjects required for this study are 215 patients with the confidence interval of 95% and margin of error of 5%.

### Measurements

#### Mini-International Neuropsychiatric Interview

The Mini-International Neuropsychiatric Interview (MINI) is a short structured diagnostic interview for a significant psychiatric disorder. Section K in the MINI was used to screen the participants to ensure they fulfil the inclusion criteria of ATS-use disorder. The MINI is a widely used instrument for research purposes (29). Moreover, the MINI is available in Malay version, which is validated in Malaysia (30).

#### Hospital Anxiety and Depression Scale

The Hospital Anxiety and Depression Scale (HADS) is a self-reported questionnaire developed by Zigmond and Snaith (31). The concurrent validity of HADS had been established among young subjects, elderly subjects, subjects with general medical illness and psychiatric patients (32). Besides, the HADS-Malay version has been proven as a reliable and valid tool to measure anxiety and depression among Malaysians (33). The Cronbach’s alpha for HADS-Malay version is 0.88 for Anxiety subscale and 0.79 for Depression subscale indicate good internal consistency. Test-retest intraclass correlation coefficient (ICC) is 0.35 and 0.42 for Anxiety and Depression subscale, respectively (33). The cut-off score of 8 was found to be appropriate for the Malaysian population with a sensitivity of 93.2% and specificity of 90.8% for both anxiety and depression (34). Therefore, this study used the scores of 8 points as the cut-off point for anxiety and depression.

### Duke University Religion Index

The Duke University Religion Index (DUREL) a self-rated scale, consisting of five questions to measure the religiosity of subjects in the study population. The DUREL includes one question to measure organised religious activity (ORA), one question to measure non-organised religious activity (NORA), and three questions to measure intrinsic religiosity (IR) (35). The ORA is referred to communal religious activities and measured by the frequently of attending religious activities in a religious institution. Meanwhile, the NORA is referred to personal and private religious activities such as reading religious books, meditation, personal religious prayer at home. Finally IR is referred to the degree of personal internal religious commitment and motivation. More than 100 publications have used DUREL, which has been translated into more than ten languages with good validation and reliability (36). Moreover, there is a translated Malay version of DUREL, DUREL-M (Duke University Religion Index-Malay version), which has been validated for the Malaysian population with good internal reliability of 0.8 (37). The lowest score for both ORA and NORA is 1 and the highest score is 6. As for IR the lowest score is 3 and the highest score is 15. The higher the score indicate higher religiosity.

### Brief RCOPE

Brief RCOPE is a self-rated questionnaire of 14 questions developed by Pargament to measure the uses of religion as a coping strategy when the study subjects face difficulty, struggles and negative life events (38). The Brief RCOPE scale can be used to measure religious coping in a multidimensional manner that is more appropriate in reflecting the essence of religious functions. Therefore, the Brief RCOPE divided religious coping into positive religious coping and negative religious coping. This scale was translated into the Malay language by Yusoff et al. (39) with high reliability as the Cronbach alpha for positive religious coping was 0.87 and the Cronbach alpha for negative religious coping was 0.88. This Brief RCOPE consists of seven items to measure the positive religious coping and negative religious coping each. The lowest scores for both positive religious coping and negative religious coping are seven with the highest score of 28. The higher the score indicate higher usage of religious coping.

## Statistical Analysis

The Statistical Package for Social Science (SPSS) version 23.0 was used to analyse the data. The independence variables (biodemographic data, religiosity and religious coping) and the dependent variables (anxiety and depression) were summarised by using descriptive statistics. The normality of the data was tested using Kolmogorov-Smirnov and Shipiro-Wilk analysis. Chi-square test was used to analyse the association of the independent and dependent variables. Subsequently, the association was further adjusted for possible confounders in a multivariate analysis by using logistic regression to ascertain more precious independence association between the variables with anxiety and depression. All the subscale for religiosity and religious coping are adjusted (polysubstance use and underlying psychiatric disorder) in multivariate analysis. The Nagelkerke R-squared for the multivariate analysis were between 0.132 to 0.271 which were relative low. All analyses were two-tailed with the alpha level of 0.05.

## Result

A total of 215 ATS users participated in this study. Table 1 displayed the sociodemographic data of the subjects. The average age of the subjects was 40.34 years, the majority of the study subjects were male (94%, *n* = 202) and Malays (61.4%, *n* = 132) and most of the subjects were Muslims (65.5%, *n* = 141). Moreover, more than half of the subjects were polysubstance users (56.3%, *n* = 121), 22.8% (*n* = 49) of the subjects had an underlying medical disorder, and 25.6% (*n* = 55) of the subjects had a psychiatry disorder.

The total mean of the subjects’ HADS score was 14.16, with a standard deviation of 8.21. The mean score for the subscale in HADS for anxiety was 6.79, with a standard deviation of 4.52. Among the 215 subjects, 44.6% (*n* = 96) patients have anxiety symptoms based on the cut-off point of 8 and above (34). Meanwhile, the mean score for the subscale of depression in HADS was 7.38, with a standard deviation of 4.40. Similarly, by using the cut-off scores of 8 and above, 50.2% (*n* = 108) of the subjects were found to be depressed.

In the analysis of the association of sociodemographic data with anxiety and depression by using Chi-square test, polysubstance use and underlying psychiatry disorder were significantly associated with both anxiety and depression (HADS scores of 8 and above for both anxiety and depression). The subjects with polysubstance use had 2.78 higher odds of having depression and 2.60 higher odds of having anxiety symptoms. Meanwhile, patients with an underlying psychiatric disorder also had 2.58 higher odds of developing depression and 2.20 higher odds of having anxiety symptoms (Table 2).

The mean score for total religiosity was 20.01 (max = 27). Majority of the participants have regular ORA with a mean score of 4.26 (max = 5) and the mean score for NORA was 3.87. Meanwhile, the mean score IR for participants was 11.88 (max = 15). Furthermore, the mean score for positive religious coping was 20.35 (max = 28), and the mean score for negative religious coping was only 12.62, which indicated that the subject used less negative religious coping as compared to positive religious coping (Table 3).

The results of univariate logistic regression analysis and adjusted multivariate logistic regression showed that all three domains of religiosity were associated with lower anxiety and depression. Similarly, positive religious coping and negative religious coping also showed significant association with anxiety and depression in both single logistic regression and multivariate logistic regression (Table 4).

## Discussion

This study demonstrated a high prevalence of anxiety (44.7%) and depression (50.2%) among 215 ATS users who sought treatment in Universiti Malaya Medical Centre, Malaysia. The results from this study support the findings of earlier studies in which ATS users had a higher risk of developing psychiatric comorbidities (40, 41). Vorpan el al. (42) also reported that depression, suicide and anxiety were the most common forms of psychopathology associated with ATS use. Meanwhile, in a study conducted among ATS users in Malaysia, Sulaiman et al. found that 16.8% of the subjects were depressed and 9.3% of the subjects had an anxiety disorder. Our study showed a higher prevalence of anxiety and depression, which could be explained due to a variety of reasons. Among them are the majority of our participants came from the psychiatry clinic, which required admission to the psychiatric ward. Meanwhile, the majority of the study subjects from Sulaiman et al.’s study were from a drug rehabilitation centre where most of them were relatively stable (28). Similarly, an epidemiological survey and meta-analysis that reviewed 115 articles demonstrated an association between substance use and major depression with the pooled odds ratio of 3.80 (95% CI = 3.02–4.78). It also showed that substance user are three times more likely to have any anxiety disorder (OR 2.91, 95% CI = 2.58–3.28). Therefore, our findings were similar to most of the data in the literature (43). Most of the research focused on more serious health morbidities, such as psychosis. However, at the same time, the psychological distress such as depression and anxiety should not be ignored as it could lead to the dreaded outcome of suicide (44).

Multiple scholars have attempted to explain the possible reasons for the high prevalence of anxiety and depression among ATS users. ATS-induced neurotoxicity is often hypothesised to cause ATS use-associated psychopathology (45). Furthermore, social-environmental factors play a role in precipitating the anxiety and depression in ATS users (46). Self-medication has been hypothesised to be a link between ATS use with anxiety and depression (47, 48). A depressed or anxious patient may try to overcome his or her difficulty by using ATS (49). The opposing view is that ATS use itself could induce anxiety disorder during intoxication, while depression is commonly observed in ATS users during withdrawal phase (42). Hence, it can be summarised that ATS use, anxiety and depression are interrelated in a complex manner (44, 50). Our results show that there is lower anxiety and depression rate among study subjects with higher religiosity. These findings are similar to those reported in the literature where religiosity is associated with a better outcome in terms of psychological well-being in a substance user. A study conducted among 1,240 adolescents demonstrated that religiosity was associated with lower psychological distress (54). Religiosity prevented anxiety and depression by having a buffering effect as it reduces the impact of the psychosocial stressor among substance users (55). The buffering occurs because the religiosity could influence a person attitudes, values and gives them meaning and purpose in life. Through cognitive and attitudinal mechanisms, the impact of psychosocial stressors would lessen and the number of substance use could reduce (56). However, there are some contradictory data in the literature where Lamb et al. (57) reported that religiosity was positively associated with higher psychological distress. This may be related to the internalisation of guilt as drug use behaviour is against religious beliefs (57).

Religious coping is becoming an increasingly popular coping tool to deal with psychological distress as religion may be able to give a sense of peace and fulfilment (58). Religious coping are further classified into positive and negative religious coping (59). This study demonstrated that positive religious coping was beneficial for ATS users’ mental health. These findings are consistent with most of the studies in the literature that focused on cancers patients (60), end-stage renal failure patients (61), and patients with a major psychiatric disorder (52). Therefore, positive religious coping could be a protective factor for patients that suffered from different forms of medical disorder, terminal illness and psychiatric disorder. In substance users, Tepper et al. (24) noticed 80% of ATS users in their study population practised some form of religious activities to deal with their daily conflicts, distresses or difficulties. The beneficial effect of positive religious coping in an ATS user could be due to the fruitful relationship with God, leading to adaptive coping mechanisms. Positive religious coping could help ATS users to deal with self-rejection and psychological distress by empowering them with positive self-image and better confidence levels by incorporating religious beliefs, values and practices (18, 19). ATS users may consider the psychosocial stressor that they encounter as a test from God and may view it as an acceptable and solvable challenge (62), which leads to lower anxiety and depression.

The study subjects demonstrated moderate to high religiosity with the mean score of 20.01 but the standard deviation is 4.96 which is very high. There is no study that assess the religiosity among ATS users in Malaysia. Nevertheless, there were multiple studies uses DUKE to measure the religiosity in Malaysian. Most of studies have the similar mean score in religiosity for Malaysia population. For example, 19.54 ± 1.69 for medical student (51), 18.29 ± 0.89 for nursing student (37), 20.52 ± 5.40 for psychiatric patients (52) and 22.35 ± 2.33 for parents with transfusion dependent thalassemia patient (53). But the standard deviation for both ATS user and psychiatric patient were higher as compared to others. These may reflect that the religiosity of this study subjects were distributed in a more extreme manner.

Meanwhile, our study also found that negative religious coping was associated with poorer psychological outcome. Negative religious coping may occur when the ATS user blames their adverse life events, or substance use behaviour was due to punishment by God and their negative relationship with God. In the case of a person who is already struggling and dissatisfied with their religious belief, the religious belief may not act as a resource for handling their emotion, but he or she may be seen as weak, distant or uncaring which may worsen their mental health (60). Therefore, negative religious copings in ATS user are rather distressing than protective (38).

The cross-sectional nature of this study limits the ability to establish the causal relationship between anxiety and depression with religiosity and religious coping. Furthermore, a single-centre study with a relatively low number of study subjects limits the generalisability. However, the study subjects consist of different races and religions as the study was conducted in a university hospital, which is a tertiary referral centre with patients coming in from various parts of the country. Another weakness of this study is that some potential confounding factors, such as the duration of ATS use, amount of ATS use, current treatment and their family support, were not collected in this study. Family plays a major role in ATS users’ mental health as strong family support and conducive environment may help in promoting better mental health in ATS user. Religion is not simplistic but rather abstract and subjective. It includes many aspects of belief, practise and living. There is diversity in the Malaysia population; the interpretation and construct of religiosity may differ across various religions. Although the psychometric properties of the measurement scales in this study were established, the scales may not be reflective and adequately measure religiosity. Formative qualitative research among ATS users in a local setting concerning their beliefs, construct, the practice of religion concerning their lives, mood and emotion can be considered in the future to better our understanding into this issue.

## Conclusion

In conclusion, this study showed a significant association between religiosity, religious coping with anxiety and depression in ATS users. Depression and anxiety are more common in ATS users with lower religiosity. The current study also showed that anxious and depressed ATS users were using more negative religious coping and not positive religious coping. Therefore, religious coping might become potentially valuable as an alternative treatment option to overcome anxiety and depression among ATS users. Implementation of religiosity and religious coping in the treatment plan of ATS users may be helpful, especially when dealing with anxious and depressed ATS users.

## Figures and Tables

**Table 1:** Sociodemographic data of the subjects (*n* = 215)

Biodemographic variables	Mean (SD)	*n* (%)
Age	40.34 (10.38)	
Gender		
Male		202 (94.0)
Female		13 (6.0)
Ethnicity		
Malay		132 (61.4)
Chinese		53 (24.7)
Indian		30 (14)
Others		0 (0)
Religion		
Islam		141 (65.6)
Buddhism		24 (11.2)
Hindu		23 (10.7)
Taoism		11 (5.1)
Christian		16 (7.4)
Education Level		
Never		0 (0)
Primary school		31 (14.4)
Secondary school		152 (70.7)
College/University		32 (14.9)
Marital status		
Single		113 (52.6)
Married		69 (32.1)
Divorced		30 (14.0)
Stable partner		3 (1.4)
Employment		
Yes		137 (63.7)
No		78 (36.3)
Monthly Income		
Less than RM1000		67 (31.2)
RM1001 to RM2000		50 (23.3)
RM2001 to RM3000		39 (18.1)
RM3001 to RM5000		10 (4.7)
More than RM5001		4 (1.9)
Not keen to disclose		45 (20.9)
Medical disorder		
Yes		49 (22.8)
No		166 (77.2)
Psychiatry disorder		
Yes		55 (25.6)
No		160 (74.4)
Polysubstance use		
Yes		121 (56.3)
No		94 (43.7)
Family history of substance use		
Yes		31 (14.4)
No		184 (85.6)
Family history of psychiatric disorder		
Yes		21 (9.8)
No		194 (90.2)

**Table 2:** Analysis of the association between biodemographic characteristic with depression (based on HADS scores > 7) among the study subjects using Chi-square test

	Depression		X^2^	OR	*P*-value	95 % CI

	Negative, *n*	Positive, *n*				
Age (years)						
Below 40	49	58	1.345	1.37	0.25	0.80–2.35
40 and above	58	50				
Gender						
Male	101	101	0.072	1.17	0.86	0.28–2.64
Female	6	7				
Ethnicity						
Malay	64	68	0.225	1.14	0.635	0.66–1.98
Non-Malay	43	40				
Religion						
Islam	60	72	0.113	1.10	0.74	0.52–1.59
Non-Islam	38	36				
Marital status						
Single	55	58	0.114	1.10	0.74	0.53–1.56
Married	52	50				
Employment						
Yes	70	67	0.864	1.16	0.61	0.50–1.51
No	67	41				
Monthly income						
RM1000 or less	33	34	0.279	1.18	0.60	0.46–1.57
More than RM1000	55	38				
Medical disorder						
Yes	22	27	0.602	1.29	0.438	0.68–2.44
No	85	81				
Psychiatry disorder **						
Yes	18	37	8.584	2.57**	0.005	1.35–4.91
No	89	71				
Polysubstance used**						
Yes	47	74	13.21	2.78**	<0.001	1.59–4.85
No	60	34				
Family history of substance use						
Yes	15	16	0.028	1.07	1.00	0.50–2.28
No	92	92				
Family history of psychiatry disorder						
Yes	10	11	0.043	1.10	1.00	0.44–2.71
No	97	97				

	**Anxiety**		**X^2^**	**OR**	***P*-value**	**95 % CI**

	**Negative, *n***	**Positive, *n***				

Age (years)**						
Below 40	49	58	9.516	2.37**	0.002	1.36–4.11
40 and above	72	36				
Gender						
Male	117	85	3.659	0.323	0.08	0.96–1.08
Female	4	9				
Ethnicity						
Malay	73	59	0.132	1.11	0.78	0.64–1.93
Non-Malay	48	35				
Religion						
Islam	78	63	0.153	1.12	0.77	0.63–1.98
Non-Islam	43	31				
Marital status						
Single	64	49	0.012	0.97	1.00	0.57–1.66
Married	57	45				
Employment						
Yes	77	60	0.001	1.01	1.00	0.57–1.77
no	44	34				
Monthly income						
RM1000 or less	33	34	1.632	1.50	0.21	0.80–2.78
More than RM1000	61	42				
Medical disorder						
Yes	24	25	1.374	1.46	0.255	0.77–2.78
No	97	69				
Psychiatry disorder^*^						
Yes	23	32	6.281	2.20^*^	0.018	1.18–4.16
No	98	62				
Polysubstance use**						
Yes	56	65	11.24	2.60**	0.001	1.48–4.58
No	65	29				
Family history of substance use						
Yes	18	13	0.047	0.92	0.85	0.43–1.98
No	103	81				
Family history of psychiatry disorder						
Yes	11	10	0.144	1.19	0.818	0.48–2.94
No	110	84				

Notes:

** *P* < 0.01

OR = odds ratio; CI = confidence interval; depression = HADS-depression subscale scores 8 and above; anxiety = HADS subscale scores 8 and above

**Table 3:** The mean scores for religiosity and religious coping (*n* = 215)

	Minimum	Maximum	Mean	SD
ORA	1	6	4.26	1.41
NORA	1	6	3.87	1.58
IR	3	15	11.88	2.73
TR	6	27	20.01	4.96
P RCOPE	7	28	20.35	5.44
N RCOPE	7	27	12.62	4.94

Notes: ORA = organised religious activity; NORA = non-organised religious activity; IR = intrinsic religiosity; TR = total religiosity; P RCOPE = positive religious coping; N RCOPE = negative religious coping

**Table 4:** Univariate and multivariate analysis of the association between religiosity, religious coping with depression and anxiety

	Depression^1^			Anxiety^2^		
	
	Crude OR	Adjusted OR	95 % CI	Crude OR	Adjusted OR	95 % CI
ORA	1.427	1.332**	1.08–1.64	1.617	1.540**	1.24–1.92
NORA	1.614	1.520**	1.25–1.87	1.481	1.379**	1.14–1.68
IR	1.418	1.388**	1.22–1.58	1.357	1.314**	1.16–1.48
TR	1.209	1.188**	1.11–1.27	1.194	1.172**	1.10–1.25
P RCOPE	1.149	1.113**	1.08–1.22	1.090	1.058*	1.00–1.12
N RCOPE	0.889	0.882**	0.84–0.95	0.863	0.862**	0.81–0.91

Notes:

* *P*-values < 0.05;

1 Depression based on: HADS sub scale scores 8 and above;

2 anxiety based on HADS subscale scores eight and above;

** *P*-values < 0.01

CI = confident interval; OR = odd ratio; Adjusted odds ratio (adjusted for psychiatric illness, poly-substance and age); ORA = organised religious activity; NORA = non-organised religious activity; IR = intrinsic religiosity; TR = total religiosity; P RCOPE = positive religious coping; N RCOPE = negative religious coping
